# The Fisher Case

**Published:** 1876-09

**Authors:** 


The Fisher Case.—We give a brief *resume* of the facts of this case, which many of our readers will recognize as the one recently referred to where death occurred during the administration of ether. The patient, a young school teacher, had suffered for two years or more from dysmenorrhœa, and had been treated by a number of physicians without relief. A few months since she placed herself under the care of Dr. Sinclair, who recommended an incision of the os, with the hope of thus effectually relieving a painful and obstinate affection. The operation was postponed until the summer vacation, when the patient would be able to give a proper amount of time to convalescence. Accordingly, early in July the patient was seen again, but was advised to wait until the approaching catamenia had passed. They having ceased on the 15th, the operation was performed at the private hospital of Mrs. Ware, on the 19th. Ether was administered, and the patient was then placed in Sims' position, the ether towel now being intrusted to a female attendant. Dr. Vogel, who assisted Dr. Sinclair at the operation, felt of the pulse soon after the operation had been begun, and found that it had ceased to beat. Breathing had ceased also, and in spite of all efforts made, the patient could not be resuscitated.

The first information which the profession and the public received of this case was that an inquest had been ordered for the purpose of determining, not whether a correct return had been made as to the cause of death, but whether an attempt had been made to procure an abortion. We need hardly say that the professional public attached no weight to the rumors and suspicions mysteriously circulated at that time. We shall refer, therefore, to but one or two points in the testimony given at the inquest. The autopsy, performed by Dr. Treadwell, showed, in addition to Bright's disease, a chronic pleurisy on one side of the chest, and an engorgement of the pulmonary artery, indicating that death resulted from asphyxia. The testimony on the condition of the ovaries and uterus, pointing to a miscarriage at some time previous to the operation, we refer to below. The jury found "that the said Clara T. Fisher came to her death on Wednesday, July 19, 1876, at about between the hours of eleven and twelve o'clock A.M., at No. 4 Ferdinand street, in Boston, by reason of suffocation, caused by the administration of sulphuric ether for a simple surgical operation, under the direction of Dr. A. D. Sinclair, assisted by Dr. Frederick W. Vogel; and the jury further find that, in their opinion, there was a lack of caution in said administration of ether, in not allowing a due quantity of atmospheric air to pass to the lungs of the patient during the etherization; and the jury are also of the opinion that there was a too hasty abandonment of the means for the resuscitation of the patient, and that the diseased condition of the patient may have contributed, in some slight degree, to her death."

Although it may be said that the jury could hardly arrive at any other opinion from the testimony as reported, excepting perhaps that referring to means taken for resuscitation, we feel sure that the professional verdict would exculpate a physician of Dr. Sinclair's high standing from any want of ordinary care in handling a case of this sort. The great superiority of ether over chloroform in point of safety has, we think, engendered among many physicians a certain want of appreciation of the amount of care necessary to be employed in the use of this, as indeed of any, powerful agent. A feeling of too great security, followed by the inevitable calamity, frequently places the blame where it is undeserved. It is not the individual or the agent, but custom, which is at fault. With precautions such as should always be taken, and which, undoubtedly, a large number of physicians think unnecessary, the warning signals will always be displayed in ample time to avert impending danger.—*Boston Medical and Surgical Journal.*

